# IS*1294* Reorganizes Plasmids in a Multidrug-Resistant Escherichia coli Strain

**DOI:** 10.1128/Spectrum.00503-21

**Published:** 2021-10-06

**Authors:** Yushan Pan, Tengli Zhang, Lijie Yu, Zhiyong Zong, Shiyu Zhao, Ruichao Li, Qianqian Wang, Li Yuan, Gongzheng Hu, Dandan He

**Affiliations:** a College of Veterinary Medicine, Henan Agricultural University, Zhengzhou, China; b West China Hospital, Sichuan University, Chengdu, China; c Jiangsu Co-Innovation Center for Prevention and Control of Important Animal Infectious Diseases and Zoonoses, Yangzhou University College of Veterinary Medicine, Yangzhou, China; University of Nebraska–Lincoln

**Keywords:** 16S rRNA methylase, cointegrate, IS*1294*, recombination, extended-spectrum β-lactamases, ESBLs

## Abstract

The aims of this study were to elucidate the role of IS*1294* in plasmid reorganization and to analyze biological characteristics of cointegrates derived from different daughter plasmids. The genetic profiles of plasmids in Escherichia coli strain C21 and its transconjugants were characterized by conjugation, S1 nuclease pulsed-field gel electrophoresis (S1-PFGE), Southern hybridization, whole-genome sequencing (WGS) analysis, and PCR. The traits of cointegrates were characterized by conjugation and stability assays. *bla*_CTX-M-55_-bearing IncI2 pC21-1 and nonresistant IncI1 pC21-3, as conjugative helper plasmids, were fused with nonconjugative *rmtB*-bearing IncN-X1 pC21-2, generating cointegrates pC21-F1 and pC21-F2. Similarly, pC21-1 and pC21-3 were fused with nonconjugative IncF33:A−:B− pHB37-2 from another E. coli strain to generate cointegrates pC21-F3 and pC21-F4 under experimental conditions. Four cointegrates were further conjugated into the E. coli strain J53 recipient at high conjugation frequencies, ranging from 2.8 × 10^−3^ to 3.2 × 10^−2^. The formation of pC21-F1 and pC21-F4 was the result of host- and IS*1294-*mediated reactions and occurred at high fusion frequencies of 9.9 × 10^−4^ and 2.1 × 10^−4^, respectively. Knockout of RecA resulted in a 100-fold decrease in the frequency of plasmid reorganization. The phenomenon of cointegrate pC21-F2 and its daughter plasmids coexisting in transconjugants was detected for the first time in plasmid stability experiments. IS*26*-*orf*-*oqxAB* was excised from cointegrate pC21-F2 through a circular intermediate at a very low frequency, which was experimentally observed. To the best of our knowledge, this is the first report of IS*1294*-mediated fusion between plasmids with different replicons. This study provides insight into the formation and evolution of cointegrate plasmids under different drug selection pressures, which can promote the dissemination of MDR plasmids.

**IMPORTANCE** The increasing resistance to β-lactams and aminoglycoside antibiotics, mainly due to extended-spectrum β-lactamases (ESBLs) and 16S rRNA methylase genes, is becoming a serious problem in Gram-negative bacteria. Plasmids, as the vehicles for resistance gene capture and horizontal gene transfer, serve a key role in terms of antibiotic resistance emergence and transmission. IS*26*, present in many antibiotic-resistant plasmids from Gram-negative bacteria, plays a critical role in the spread, clustering, and reorganization of resistance determinant-encoding plasmids and in plasmid reorganization through replicative transposition mechanisms and homologous recombination. However, the role of IS*1294*, present in many MDR plasmids, in the formation of cointegrates remains unclear. Here, we investigated experimentally the intermolecular recombination of IS*1294*, which occurred with high frequencies and led to the formation of conjugative MDR cointegrates and facilitated the cotransfer of *bla*_CTX-M-55_ and *rmtB*, and we further uncovered the significance of IS*1294* in the formation of cointegrates and the common features of IS*1294*-driven cointegration of plasmids.

## INTRODUCTION

The emergence and dissemination of antibiotic resistance is a major clinical problem that poses a serious threat to public health (1). Antibiotic resistance genes are associated with mobile genetic elements like plasmids, transposons, and integrons (2). Among them, plasmids play a key role as vehicles for resistance gene capture and subsequent dissemination (3). Plasmid interaction is important for the maintenance and conjugal transfer of plasmids, particularly the mobilization of nonconjugative plasmids (4). The fusion of nonconjugative plasmids and conjugative helper plasmids is often related to different recombination events, namely, homologous recombination and replicative transposition, facilitating the dispersal of resistance genes and the evolution of multidrug resistance (MDR) plasmids and extending the resistance profiles of cointegrate plasmids, which has raised wide concerns (5–11).

Insertion sequences IS*26* and IS*1294* are present in many antibiotic-resistant isolates and play critical roles in the diversity of the variable region of F33:A−:B− plasmids carrying *bla*_CTX-M-55_ or *bla*_CTX-M-65_ (12). Three well-characterized fusion plasmids mediated by IS*26* have been reported in clinical strains, namely, pSL131_IncA/C_IncX3, pD72C, and pSE380T (5–7). IS*1294*, a member of the IS*91* family, is an atypical insertion sequence that lacks terminal inverted repeats, does not generate target site duplication, and transposes using rolling-circle replication (13). The IS*1294*-mediated formation of cointegrate plasmids is rarely reported. In our previous study, the *bla*_CTX-M-55_- and *rmtB*-bearing sequence type 156 (ST156) Escherichia coli strain C21 from a chicken in China was characterized, and the IS*Ecp1* element located upstream from *bla*_CTX-M-55_ was found to be disrupted by IS*1294* (14). Here, two plasmids, used as conjugative helper plasmids, were fused with the nonconjugative *rmtB*-carrying plasmid in strain C21 at high fusion frequencies, generating two conjugative cointegrates that could be further transferred into recipient E. coli strain J53 at high conjugation frequencies. Consequently, the role of IS*1294* in the formation of cointegrate plasmids was experimentally verified.

## RESULTS

### Characterization of multidrug-resistant E. coli strain C21.

Multidrug-resistant E. coli strain C21 belonging to ST156 was isolated from a chicken in China in our previous study (14). Whole-genome sequencing (WGS) indicated that strain C21 contained a 4,757,725-bp chromosome and four plasmids, designated pC21-1 (63,878 bp), pC21-2 (62,933 bp), pC21-3 (87,627 bp), and pC21-4 (93,854 bp), and carried multiple antimicrobial resistance genes (Table 1).

**TABLE 1:** Characterization of E. coli strain C21 and its transconjugants and transformants used in this study

Strain	Isolate	Plasmid	Size (bp)	Replicon type	Self-transferability	Resistance genes	Resistance phenotype^*a*^
Parental strain	C21	pC21-1	63,878	IncI2	Conjugative	*bla*_CTX-M-55_	AM, CAZ, CTX, CIP, ENR, KAN, AN, GM, DO, FFC
		pC21-2	62,933	IncN-X1	Nonconjugative	*rmtB*, *oqxAB*, *bla*_TEM-1b_, *floR*, *tet*(A), *strA*, *strB*, *sul1*, *sul2*, *aac(3)-IId*, *aadA2*, *dfrA12*, *aph(3′)-IIa*	
		pC21-3	87,627	IncI1	Conjugative	No resistance gene	
		pC21-4	93,854	IncY	Nonconjugative	No resistance gene	

Transconjugants^*b*^	TC21-1	pC21-1	63,878	IncI2	Conjugative	*bla*_CTX-M-55_	AM, CAZ, CTX
	TC21-F1	pC21-F1	126,764	IncI2-N-X1	Conjugative	*bla*_CTX-M-55_, *rmtB*, *oqxAB*, *bla*_TEM-1b_, *floR*, *tet*(A), *strA*, *strB*, *sul1*, *sul2*, *aac(3)-IId*, *aadA2*, *dfrA12*, *aph(3′)-IIa*	AM, CAZ, CTX, KAN, AN, GM, DO, FFC
	TC21-F2	pC21-F2	144,11	IncI1-N-X1	Conjugative	*rmtB*, *oqxAB*, *bla*_TEM-1b_, *floR*, *tet*(A), *strA*, *strB*, *sul1*, *sul2*, *aac(3)-IId*, *aadA2*, *dfrA12*, *aph(3′)-IIa*	AM, KAN, AN, GM, DO, FFC
	TC21-F3	pC21-F3	∼158,000	IncI1-F33:A−:B−	Conjugative	*bla*_TEM-1b_, *rmtB*	AM, KAN, AN, GM
	TC21-F4	pC21-F4	∼135,000	IncI2-F33:A−:B−	Conjugative	*bla*_CTX-M-55_, *bla*_TEM-1b_, *rmtB*	AM, CAZ, CTX, AM, KAN, AN, GM

Transformants	TC21-2	pC21-2	62,933	IncN-X1	Nonconjugative	*rmtB*, *oqxAB*, *bla*_TEM-1b_, *floR*, *tet*(A), *strA*, *strB*, *sul1*, *sul2*, *aac(3)-IId*, *aadA2*, *dfrA12*, *aph(3′)-IIa*	AM, KAN, AN, GM, DO, FFC
	TC21-3	pC21-3	87,627	IncI1	Conjugative	No resistance gene	
	THB37-2	pHB37-2	71,222	F33:A−:B−	Nonconjugative	*bla*_TEM-1b_, *rmtB*	AM, KAN, AN, GM
	TC21-1-HB37-2	pC21-1, pHB37-2				*bla*_CTX-M-55_, *bla*_TEM-1b_, *rmtB*	AM, CAZ, CTX, AM, KAN, AN, GM
	TC21-3-HB37-2	pC21-3, pHB37-2				*bla*_TEM-1b_, *rmtB*	AM, KAN, AN, GM

a AM, ampicillin; CAZ, ceftazidime; CTX, cefotaxime; KAN, kanamycin; GM, gentamicin; AN, amikacin; CIP, ciprofloxacin; ENR, enrofloxacin; DO, doxycycline; FFC, florfenicol.

b Transconjugant TC21-1 carrying *bla*_CTX-M-55_ was screened on MacConkey agar plates supplemented with rifampin and cefotaxime, transconjugant TC21-F1 carrying *bla*_CTX-M-55_ and *rmtB* was screened on MacConkey agar plates supplemented with rifampin, cefotaxime, and amikacin, and transconjugant TC21-F2 carrying *rmtB* was screened on MacConkey agar plates supplemented with rifampin and amikacin.

### Sequence analysis of plasmids in C21.

**FIG 1:**
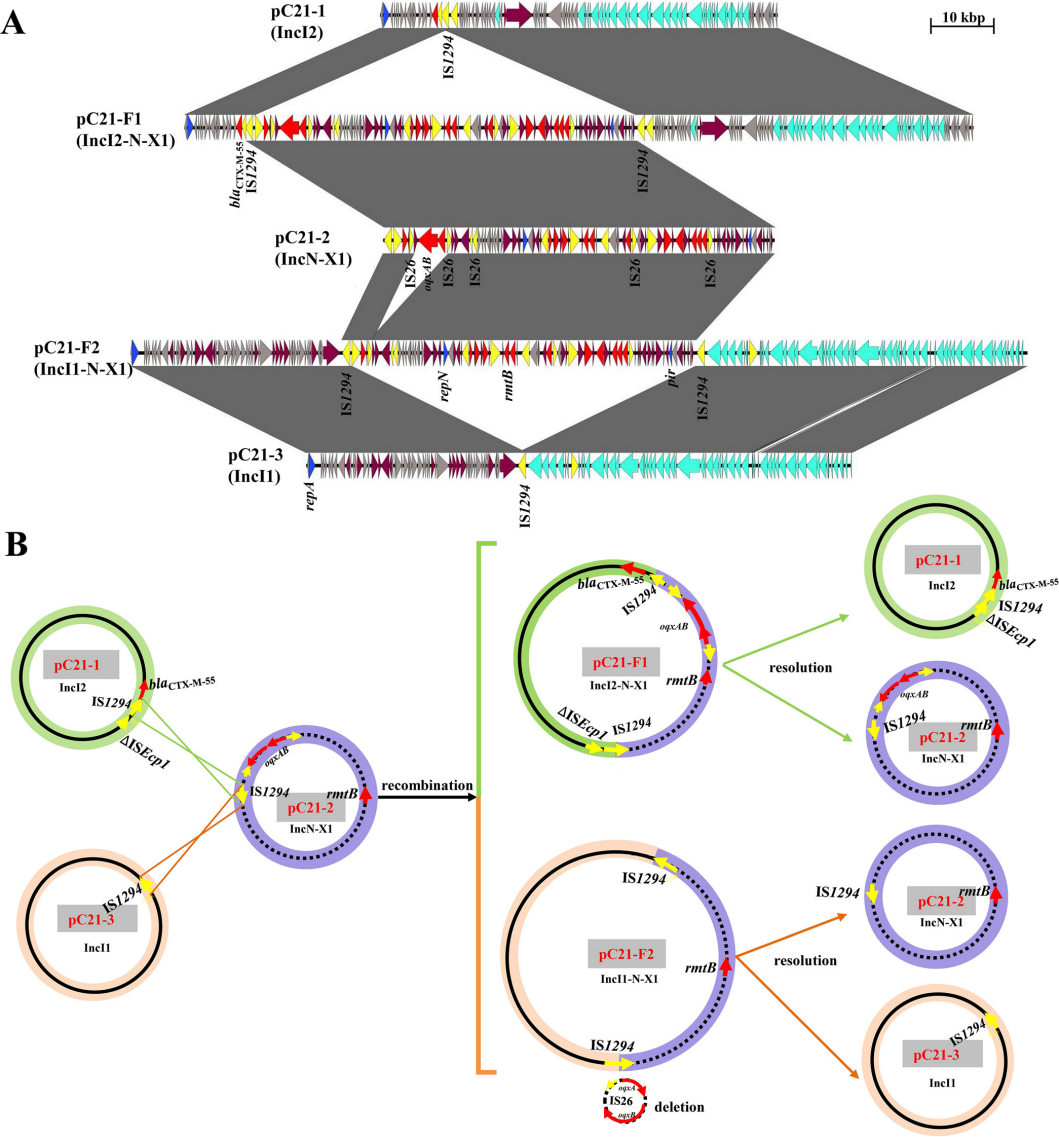
The proposed mechanism of plasmid fusion. (A) Linear sequence comparison of two fusion plasmids, pC21-F1 and pC21-F2, with daughter plasmids pC21-1, pC21-2, and pC21-3. Colored arrows represent open reading frames, with blue, cyan, red, yellow, maroon, and gray arrows representing replicon genes, transfer-associated genes, resistance genes, mobile elements, stability associated genes, and hypothetical proteins, respectively. The shaded areas indicate 100% identity. (B) The proposed model for the IS*1294*-mediated formation of fusion plasmids. Plasmid names are shown in red on a gray background. Arrowheads indicate orientation. The cointegrates were brought about by intermolecular homologous recombination. Cointegrates pC21-F1 and pC21-F2 could subsequently be resolved into two plasmids identical to the original donor plasmids except for the excision of IS*26*-*orf*-*oqxAB*. Yellow arrows represent IS elements, and gray arrows represent hypothetical proteins.

The *bla*_CTX-M-55_-positive pC21-1 harbored an IncI2 replicon and typical IncI2-associated genetic modules responsible for plasmid replication, transfer, maintenance, and stability functions. Sequence analysis revealed that pC21-1 shared high degrees of genetic identity (99 to 100% identity at 97 to 99% coverage) with several known *bla*_CTX-M_-bearing IncI2 plasmids, including pHNY2, pHN1122-1, pHNAH46-1, and pHNLDH19, in E. coli strains isolated from different sources (Fig. S1A), and the IS*Ecp1* located upstream from *bla*_CTX-M-55_ in pC21-1 differed from the IncI2 plasmids mentioned above by the insertion of an IS*1294* (Fig. 1A).

The multireplicon IncN-X1 plasmid pC21-2, with *repE* and *pir* genes, which are responsible for the replication initiation of IncN and IncX1, harbored the resistance genes *rmtB*, *oqxAB*, *bla*_TEM-1b_, *floR*, *tet*(A), *strAB*, *sul1*, *sul2*, *aac(3)-IId*, and *aph(3′)-IIa* and a class 1 integron cassette array, *dfrA12*-*orfF*-*aadA2*, as well as mobile elements, including one IS*1294* and five intact IS*26* copies with no direct repeats (DRs) (Table 1 and Fig. 1A). The fusion of segments in pC21-2 containing replication regions from the conjugative IncX1 plasmid pOLA52 in a swine E. coli strain and the classical IncN plasmid R46 in Salmonella enterica serovar Typhimurium (15, 16) might be mediated by IS*26* through homologous recombination (Fig. 1A and Fig. S1A). A BLASTN search revealed that pC21-2 exhibited high homology to the *bla*_NDM-1_-positive IncN-X1 plasmid p1108-NDM, with 99.9% identity at 81% coverage; however, the main multidrug resistance regions of pC21-2 were almost identical with those of IncI1/ST136 pEC008 (accession number KY748190) (Fig. S1B) (17). The pC21-2 plasmid, without a transfer region, was not self-transmissible, which was determined by conjugation assays showing that no transconjugant was obtained after numerous attempts using the transformant TC21-2 carrying pC21-2 as the donor.

pC21-3, without an antimicrobial resistance gene, belonged to IncI1/ST134 except for one nucleotide substitution (G→A) in the conjugative transfer gene *trbA*. BLAST analysis showed that pC21-3 exhibited 98.2 and 98.7% identity at 93 and 95% coverage with two conjugative helper plasmids, the nonresistant pSa27-HP (accession number MH884654) and the CTX-M-130-producing pSa44-CRO (accession number MH430883), recovered from Salmonella strains (8, 9). pC21-4, a phage-like IncY plasmid without any antimicrobial resistance gene, had a single pO111 plasmid replicon and exhibited high homology to p1108-IncY in E. coli (accession number MG825379), with 99% identity at 93% coverage.

### Identification of fusion plasmids.

In previous work, we showed that two important resistance determinants, *bla*_CTX-M-55_ and *rmtB*, were present in separate plasmids in strain C21 and could be cotransferred into the recipient strain (14). In this work, three representative transconjugants, TC21-1, TC21-F1, and TC21-F2, were screened successfully by conjugation experiments using different antibiotics (Table 1). S1 nuclease pulsed-field gel electrophoresis (S1-PFGE) and Southern blot hybridization confirmed that *bla*_CTX-M-55_ and *rmtB* were located on the ∼60-kb pC21-1 plasmid and the ∼60-kb pC21-2 plasmid, respectively, in the parental strain C21. However, *bla*_CTX-M-55_ coexisted with *rmtB* on a single ∼120-kb plasmid, pC21-F1, in TC21-F1, and *rmtB* was located on a single ∼140-kb plasmid, pC21-F2, in TC21-F2. The pC21-F1 and pC21-F2 plasmids were larger than any plasmid in the original strain C21 (Fig. S2A). In view of the plasmid sizes, we proposed that pC21-F1 might be the recombinant product of pC21-1 (63,878 bp) and pC21-2 (62,933 bp) and pC21-F2 might be the recombinant product of pC21-2 (62,933 bp) and pC21-3 (87,627 bp). To further probe the sources of fusion plasmids, the complete sequences of plasmids in the transconjugant strains were obtained by WGS, combining the Illumina short-read and PacBio long-read sequencing data.

### Reorganization mechanism of fusion plasmids mediated by IS*1294*.

Sequence analysis showed that the *bla*_CTX-M-55_- and *rmtB*-bearing pC21-F1 was 126,764 bp in length and the *rmtB*-bearing pC21-F2 was 144,112 bp in length; the genotypes were consistent with the phenotypes of transconjugants TC21-F1 and TC21-F2, respectively (Table 1). Further analysis showed that pC21-F1 and pC21-F2 were hybrid plasmids presenting chimeric characteristics mediated by IS*1294*. The pC21-F1 plasmid consisted of pC21-1 (nucleotides [nt] 1 to 9655; 72421 to 126764) and pC21-2 (nt 9656 to 72420), and pC21-F2 consisted of pC21-3 (nt 1 to 33895; 90871 to 144112) and pC21-2, lacking the *oqxAB* determinant (nt 33896 to 90870). Two copies of IS*1294* in the same orientation were embedded into the junction regions of the two fusion plasmids (Fig. 1).

**FIG 2:**
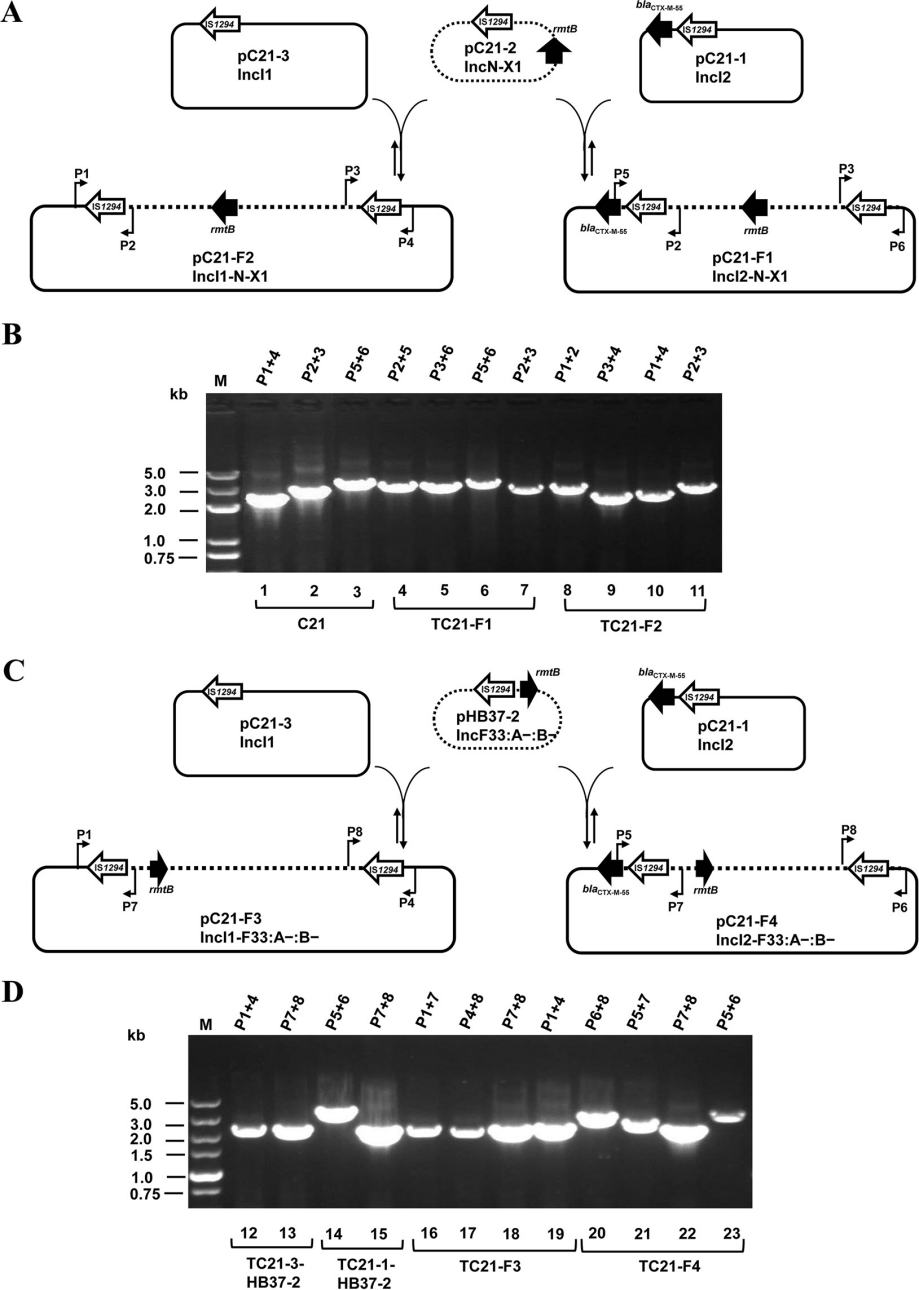
Schematic representation of the formation of fusion plasmids and the dynamic process between cointegrates and daughter plasmids in the original strain and transconjugants. (A) The regeneration and resolution of cointegrate plasmids pC21-F1 and pC21-F2. Black arrows represent antibiotic resistance genes, and hollow arrows represent IS*1294*. The dotted and solid lines represent the framework of plasmids. The locations of primers used to map targeted cointegrates are shown. Note that the figure is not to scale. (B) Gel electrophoresis of PCR amplicons corresponding to the different combinations of primers. PCR amplicons 1 to 3 were obtained from the parental strain C21, amplicons 4 to 7 were obtained from the transconjugant TC21-F1, and amplicons 8 to 11 were obtained from the transconjugant TC21-F2. (C) The regeneration and resolution of cointegrate plasmids pC21-F3 and pC21-F4. (D) Gel electrophoresis of PCR amplicons corresponding to the different combinations of primers. PCR amplicons 12 to 13 were obtained from the transformant TC21-3-HB37-2, amplicons 14 to 15 were obtained from the transconjugant TC21-1-HB37-2, amplicons 16 to 19 were obtained from the transconjugant TC21-F3, and amplicons 20 to 23 were obtained from the transconjugant TC21-F4.

Based on the sequence analysis detailed above and the observed structure, we proposed the model of cointegrate formation shown in Fig. 1B. In the model, the IS*1294* element in non-self-transmissible pC21-2 (IncN-X1) attacked another IS*1294* in the conjugative pC21-1 or pC21-3, resulting in the occurrence of cointegrates. Linearized pC21-1 or pC21-3 was incorporated into pC21-2, creating the cointegrates pC21-F1 and pC21-F2, and then, two same-orientation IS*1294* elements surrounded the insertion fragment. The sequences spanning the cointegrate junctions were confirmed using primers P1-P2 and P3-P4 for pC21-F2 and P2-P5 and P3-P6 for pC21-F1 and sequences of PCR amplicons corresponding to the result of WGS (Fig. 2A and B). A dynamic process occurred between cointegrates and daughter plasmids in transconjugants, which was identified by PCR and sequencing, and several amplicons of combinations of primers to detect the flanking sequence of IS*1294* were obtained (Fig. 2B). IS*1294* lacking terminal inverted repeats does not generate DRs of the target site and transposes by rolling-circle replication (13). Although DRs of IS*1294* surrounding the insertion fragments were not detected in this study, IS*1294*-mediated intermolecular recombination was likely to be related to the formation of cointegrates.

To analyze the lack of IS*26*-*orf*-*oqxAB* in the pC21-2 segment of the cointegrate pC21-F2, reverse PCR was performed to detect potential circular intermediates from IS*26*-*orf*-*oqxAB*-IS*26* in pC21-2 in the parental strain, transconjugants, and transformant. The minicircle (IS*26*-*orf*-*oqxAB*) containing the central region and one intact IS*26* was detected in the parental strain, transconjugant TC21-F1 (pC21-F1), and transformant TC21-2 (pC21-2) but not in transconjugant TC21-F2 (pC21-F2) (Fig. 1). However, the *oqxAB* determinant was excised at a low probability, as suggested by the detection of *oqxAB* in 80 randomly selected transconjugants screened on MacConkey agar supplemented with rifampin and amikacin.

To determine whether IS*1294*-mediated cointegration occurred in other incompatibility plasmids and how often, the 71,222-bp nonconjugative *rmtB*-bearing IncF33:A−:B− plasmid pHB37-2 with a single IS*1294* was analyzed. As expected, pC21-3 and pC21-1 could each be fused with pHB37-2, generating two cointegrates, pC21-F3 (IncI1-F33:A−:B−) and pC21-F4 (IncI2-F33:A−:B−), in transconjugants (Fig. S2B). Similar to cointegrates pC21-F1 and pC21-F2, several amplicons of combinations of primers for detecting the flanking sequence of IS*1294* in fusion regions in pC21-F3 and pC21-F4 were obtained by PCR and Sanger sequencing (Fig. 2C and D).

### IS*1294*-mediated intermolecular recombination with high frequency leads to stable conjugative MDR cointegrates.

The conjugation frequencies of cointegrates pC21-F1 and pC21-F2 were 7.1 × 10^−9^ and 2.6 × 10^−7^ transconjugants per donor, respectively. However, pC21-F1 and pC21-F2 could be further transferred from the transconjugants to the recipient E. coli J53 strain at higher conjugation frequencies of 2.8 × 10^−3^ and 3.2 × 10^−2^ transconjugants per donor, respectively (Table S2). In addition, the conjugation frequency of the parental plasmid pC21-1 was 3.6 × 10^−4^ cefotaxime-resistant transconjugants per donor. The conjugation frequencies of fusion plasmids pC21-F3 and pC21-F4 from the parental strain were 5.3 × 10^−7^ and 1.0 × 10^−7^ transconjugants per donor. pC21-F3 and pC21-F4 could be further transferred from the transconjugants to the recipient E. coli J53 at higher conjugation frequencies of 2.7 × 10^−2^ and 1.2 × 10^−2^ transconjugants per donor, respectively (Table S2).

The fusion frequency of cointegrate pC21-F1 from pC21-1 and pC21-2 was 9.9 × 10^−4^ transconjugants per cefotaxime-resistant transconjugant (Table S3). The fusion frequency of pC21-F2 could not be determined because pC21-3 did not have an antibiotic resistance marker (Table 1). However, the number of transconjugants carrying pC21-F2 from the parental strain was significantly higher than that of transconjugants carrying pC21-F1 from the parental strain in conjugation assays. Based on these data, we speculated that the fusion frequency of pC21-F2 was higher than that of pC21-F1, which was further confirmed by a conjugation assay using E. coli C21 as the donor and E. coli C600 as the recipient. The results of the assay showed that 80 randomly selected transconjugants screened by rifampin and amikacin carried the *rmtB* gene but not *bla*_CTX-M-55_. The fusion frequency of pC21-F4 (IncI2-F33:A−:B−) was 2.1 × 10^−4^ transconjugants per cefotaxime-resistant transconjugant (Table S3). Comparative assays were performed in wild-type and recombination-deficient (*ΔrecA*) donor strains, with the results showing that host- and IS*1294*-mediated reactions were involved in the formation of cointegrate plasmids and that knockout of *recA* resulted in a 100-fold decrease (from 3.0 × 10^−4^ to 4.8 × 10^−6^) in the frequency of plasmid reorganization (Table S4).

Stability assays *in vitro* showed that <10% losses of fusion plasmids pC21-F1 and pC21-F2 in transconjugants occurred from day 1 to day 15, which suggested that fusion plasmids were stable in E. coli for at least 15 days of passage in an antibiotic-free environment (Fig. S3). A total of 40 amikacin- and cefotaxime-susceptible colonies from TC21-F1 were detected among 1,800 colonies screened at 0, 3, 6, 9, 12, and 15 days (100 colonies screened at six time points in each of three independent experiments). S1-PFGE showed that randomly selected colonies with the resistant phenotype originating from TC21-F1 harbored a single fusion plasmid, pC21-F1 (data not shown), suggesting that the fusion plasmid pC21-F1 was not easily lost and cleaved. However, 123 amikacin-susceptible colonies from TC21-F2 were detected among 1,800 colonies screened, and 2 of 14 colonies carried the daughter plasmids at 12 and 15 days (Fig. S4). As shown in the electrophoretic bands of lane 1 presented in Fig. S4B, the fusion plasmid pC21-F2 and its daughter plasmids coexisted in transconjugant TC21-F2 at 12 days, suggesting that the cointegrate and daughter plasmids may be in a dynamic process.

## DISCUSSION

In an exploration of the evolutionary process of F33:A−:B− plasmids, Wang et al. found that several IS*26* and IS*1294* elements were interspersed in MDR regions of F33:A−:B− plasmids carrying *bla*_CTX-M-55_ or *bla*_CTX-M-65_, causing diversity in the variable regions of the plasmids (12). The IS*26*-mediated formation of fusion plasmids in transconjugants has been well described in the hybrid resistance plasmid pD72C and the virulence and resistance plasmid pSE380T (5, 7). However, the IS*26*-mediated fusion plasmid pSL131_IncA/C_IncX3 was identified in the parental strain, and its daughter plasmid pSL131T_IncX3 carrying *bla*_NDM-1_ was detected in the corresponding transconjugant (6). The IS*Pa40*-mediated fusion plasmid pSa44-CIP-CRO was also illustrated in the parental strain, and two corresponding transconjugants selected in eosin methylene blue agar supplemented with different agents harbored the fusion plasmid pSa44-CIP-CRO and its daughter plasmid pSa44-CRO (8). In the present study, three transconjugants were obtained from the parental strain C21 under selective pressure by different agents; one of them carried a daughter plasmid, and the other two carried different fusion plasmids mediated by IS*1294*. Two cointegrates, pC21-F1 and pC21-F2, were not observed in the parental strain by S1-PFGE and complete sequencing; however, a dynamic process occurred between cointegrate and daughter plasmids in the transconjugants. The different states in the cointegrate plasmids between the original strain and transconjugants may be due to the abundance of cointegrate plasmids. Although the cointegrate plasmids may be in low abundance in the original strain harboring daughter plasmids, they were in high abundance after antibiotic drug selection. Taken together, the findings indicated that the cointegrate plasmid was easily selected and disseminated under pressure by different agents. Furthermore, the cointegrate plasmids mediated by IS elements were ubiquitous, and the replicon typing of the daughter plasmids from fusion plasmids was diverse. Studies have demonstrated that the cointegrates were formed between two DNA molecules in a process mediated by IS*26* through a replicative transposition mechanism (7, 18, 19). However, in the present study, IS*1294*-mediated intermolecular recombination was involved in the formation of cointegrates.

The differences in the abundance of fusion plasmids pC21-F1 and pC21-F2 between the parental strain and transconjugants were consistent with their conjugation frequencies. A 4 × 10^5^-fold increase in the conjugation frequency of pC21-F1 from transconjugant TC21-F1 to recipient E. coli J53 was noted when compared with the conjugation frequency of pC21-F1 from the parental strain C21 to recipient E. coli C600 (from 7.1 × 10^−9^ to 2.8 × 10^−3^), and a 1.2 × 10^5^-fold increase in the conjugation frequency for pC21-F2 was noted (from 2.6 × 10^−7^ to 3.2 × 10^−2^). Similar conjugation frequency results were obtained for cointegrate plasmids pC21-F3 and pC21-F4 (Table S2). These findings indicated that pC21-1 and pC21-3 may act as conjugative helper plasmids, providing nonconjugative plasmids pC21-2 (IncN-X1) and pHB37-2 (IncF33:A−:B−) with self-transmission capacity through the formation of cointegrates. In addition, their activity may lead to the rapid transmission of resistance genes in nonconjugative plasmids under selection by antibiotics, as well as promoting the evolution of MDR plasmids.

The average fusion frequencies were 9.9 × 10^−4^ and 2.1 × 10^−4^, respectively, for cointegrates pC21-F1 and pC21-F4, which resulted from host-mediated homologous recombination and IS*1294*-mediated intermolecular reactions. Comparison analysis performed in the wild-type and recombination-deficient (*ΔrecA*) donor strains showed that knockout of *recA* resulted in a 100-fold decrease (from 3.0 × 10^−4^ to 4.8 × 10^−6^) in the fusion frequency of cointegrate pC21-F1, which suggested that IS*1294*-mediated reactions, with an average transposition frequency of 4.8 × 10^−6^ for pC21-F1, and intrinsic homologous recombination played major roles in plasmid reorganization. The frequency of cointegrate formation mediated by IS*26* between pRMH762 and the construct R388::IS*26* was 1.8 × 10^−4^ per R388::IS*26* transconjugant in transposition experiments (18). The high fusion efficiency mediated by IS*1294* or IS*26* highlighted the important role of IS*1294* and IS*26* in the generation of cointegrate plasmids and the dissemination of resistance genes.

In summary, this study characterized the complete genetic features of four plasmids and elucidated the mechanism underlying the reorganization of fusion plasmids. To the best of our knowledge, this is the first description of the role of IS*1294* in the formation of fusion plasmids derived from three plasmids in an original strain. This study provided insight into the formation and evolution of cointegrates under the selective pressure of one or more antimicrobials, which poses a serious threat to public health. Therefore, more prudent use of antimicrobial agents in clinical practice, particularly the use of antibiotic combinations, is important to avoid the occurrence, dissemination, and further evolution of MDR fusion plasmids.

## MATERIALS AND METHODS

### Bacterial strain.

Multidrug-resistant ST156 E. coli strain C21, carrying two important resistance determinants, *bla*_CTX-M-55_ and *rmtB*, in separate plasmids was characterized from a chicken in China in September 2009 as described in our previous study (14).

### Antimicrobial susceptibility testing.

The MICs of the parental strain, corresponding transconjugants, and transformants against 11 antibiotics, including ampicillin, cefotaxime, ceftazidime, cefoxitin, amikacin, gentamicin, doxycycline, florfenicol, enrofloxacin, ciprofloxacin, and colistin, were determined using the broth microdilution method and interpreted in accordance with CLSI standards (20). The criterion for florfenicol was interpreted according to EUCAST (http://mic.eucast.org/Eucast2/). E. coli strain ATCC 25922 was used as the quality control strain.

### Conjugation, transformation, S1-PFGE, and Southern hybridization.

E. coli C21 as the donor and E. coli C600 (resistant to rifampin) as the recipient were used in conjugation experiments. Three representative transconjugants were screened on MacConkey agar supplemented with rifampin (450 mg/liter), cefotaxime (2 mg/liter), and/or amikacin (20 mg/liter). The conjugation frequencies were calculated as the number of transconjugants per donor. The plasmids in the donor strain C21 were transformed into E. coli DH5a by electroporation; the *rmtB*-bearing transformant TC21-2 was selected on LB agar supplemented with amikacin (20 mg/liter), and transformant TC21-3, harboring a single pC21-3 without any antibiotic resistance genes, was selected on antibiotic-free LB agar. Plasmid profiles in the donor strain, transconjugants, and transformants were subjected to S1-PFGE and Southern blot hybridization with *bla*_CTX-M-55_, *rmtB*, and *trbA* for the IncI1 plasmid as probes.

### WGS and bioinformatics analysis.

To explore the genetic basis of plasmid size alteration in the donor and transconjugant strains, total genomic DNA was extracted from C21 and the plasmids in transconjugants TC21-F1 and TC21-F2 using the Omega bacterial DNA kit (Omega Bio-Tek, USA) and the Qiagen plasmid midi kit (Qiagen, Hilden, Germany) and subjected to whole-genome sequencing (WGS) using Illumina NovaSeq 6000 and the PacBio RSII single-molecule real-time (SMRT) platforms. The long-read data were assembled *de novo* using the hierarchical genome assembly process (HGAP) with the SMRT Analysis version 2.3.0 software package for the PacBio RSII platform, in combination with complementary short reads (21). The plasmid sequences were initially annotated using the Subsystem Technology (RAST version 2.0) server (http://rast.nmpdr.org) and curated manually using the BLASTn and BLASTp algorithms (http://blast.ncbi.nlm.nih.gov/blast). The plasmid replicon genotype and resistance genes were identified by using the CGE server (https://cge.cbs.dtu.dk/services/). The comparative analysis and plasmid maps were generated using Easyfig and BRIG (22, 23).

### Identification of circular intermediates carrying *oqxAB*.

Reverse PCR was performed to detect the potential circular form of the IS*26*-flanked transposon carrying *oqxAB* in the parental strain C21, transconjugants, and transformants. PCR with TaKaRa *Taq* DNA polymerase was carried out with an initial denaturation at 94°C for 5 min, followed by 30 cycles of amplification (denaturation at 94°C for 30 s, annealing at 57°C for 30 s, and extension at 72°C for 2 min) and a final extension at 72°C for 10 min. To further assess the excision of IS*26*-*orf*-*oqxAB*-IS*26*, a conjugation assay was performed under rifampin and amikacin selection, and *oqxAB* was identified in 80 randomly selected transconjugants by PCR using the *oqxAB*-F/R primers listed in Table S1.

### Recombination and conjugation frequencies of fusion plasmids.

To investigate the ability to form the fusion plasmid pC21-F1 from conjugative *bla*_CTX-M-55_-positive pC21-1 (IncI2) and nonconjugative *rmtB*-positive pC21-2 (IncN-X1), recombination frequencies were identified by conjugation assay using strain C21 as the donor and E. coli C600 as the recipient. The recombination frequency was calculated as the number of transconjugants carrying fusion plasmid pC21-F1 per cefotaxime-resistant transconjugant. The recombination frequency for the fusion plasmid pC21-F2 from conjugative pC21-3 (IncI1) and pC21-2 could not be determined because of the lack of a selective marker for pC21-3.

To explore the role of IS*1294* in plasmid reorganization, a comparative analysis between wild-type and recombination-deficient (*ΔrecA*) donor strains was performed. Both pC21-2 and pC21-1 were transformed into E. coli C600 and recombination-deficient (*ΔrecA*) E. coli C600, respectively, generating two corresponding transformants, and then the transformants as the donor and E. coli J53 (*ΔrecA*) as the recipient were used in conjugation experiments. Transconjugants carrying cointegrate pC21-F1 were selected on LB agar plates supplemented with cefotaxime (2 mg/liter) and amikacin (20 mg/liter). All the transformants and transconjugants were confirmed by the presence of *bla*_CTX-M-55_, *rmtB*, and fusion points by PCR and Sanger sequencing. Recombination frequencies were calculated as the number of transconjugants carrying pC21-F1 per cefotaxime-resistant transconjugant.

To confirm plasmid reorganization *in vitro*, the E. coli DH5a transformant THB37-2 carrying the nonconjugative *rmtB*-positive pHB37-2 with one IS*1294* copy was recruited. Reorganizations of pHB37-2 (IncF33:A−:B−) with pC21-1 (IncI2) and pC21-3 (IncI1) were performed using the following method: pC21-1 was transformed into THB37-2, and the corresponding transformant, designated TC21-1-HB37-2, was selected on LB agar plates supplemented with cefotaxime (2 mg/liter) and amikacin (20 mg/liter). Similarly, pHB37-2 was transformed into the E. coli DH5a transformant TC21-3, and the corresponding transformant was designated TC21-3-HB37-2. Then, transformants were used as the donors for conjugation experiments with E. coli C600 as described above. The recombination frequency was calculated as described above.

To assess the self-transferability of the fusion plasmids pC21-F1 and pC21-F2 in transconjugants, conjugation assays were further performed using E. coli C600 transconjugants TC21-F1 and TC21-F2 as the donor and azide-resistant E. coli J53 as the recipient, and conjugation frequencies were calculated as the number of transconjugants per donor. All the transformants and transconjugants were verified by PCR and antimicrobial susceptibility testing. Plasmid profiles in the transconjugant and transformant strains were subjected to S1-PFGE and Southern blot hybridization, and the fusion points were detected by PCR and sequencing. The sequences and approximate positions of the primers are shown in Table S1 and Fig. 2A and C.

### Plasmid stability.

The stability of fusion plasmids pC21-F1 and pC21-F2 was assessed as described previously (24). In brief, transconjugants TC21-F1 and TC21-F2 were propagated by serial transfer for 15 days of passage. The culture broths were serially diluted in 0.85% saline and plated onto antibiotic-free LB agar at 0, 3, 6, 9, 12, and 15 days. A total of 100 colonies were randomly chosen and plated onto LB agar supplemented with amikacin and cefotaxime for TC21-F1 and with amikacin for TC21-F2, and then PCR was performed to confirm the presence of *bla*_CTX-M-55_ and *rmtB* in TC21-F1 colonies and *rmtB* and IncI1 replicon types for TC21-F2 colonies. The numbers of colonies were calculated at six time points in each of three independent experiments. The plasmid profiles of 14 randomly selected colonies from TC21-F1 or TC21-F2 were further identified using S1-PFGE. In all instances, the patch counts were consistent with the colony counts.

### Data availability.

The complete sequences of the chromosome and four plasmids in strain C21 and fusion plasmids pTC21-F1 and pTC21-F2 in two transconjugants were submitted to GenBank with the following accession numbers: chromosome (CP052877), pC21-1 (CP052878), pC21-2 (CP052879), pC21-3 (CP052880), pC21-4 (CP052881), pC21-F1 (MT554516), and pC21-F2 (MT554517). The complete sequence of nonconjugative *rmtB*-bearing pHB37-2 was also submitted to GenBank with the accession number CP053082.
