# Supplementary material for: IS1294 Reorganizes Plasmids in a Multidrug-Resistant Escherichia coli Strain
Source: Microbiol Spectr. 2021 Oct 6;9(2):e00503-21. doi: 10.1128/Spectrum.00503-21 (PMC8510248; doi:10.1128/Spectrum.00503-21)
Supplement: SUPPLEMENTAL FILE 1 — Supplemental material. Download SPECTRUM00503-21_Supp_1_seq4.pdf, PDF file, 0.7 MB [file spectrum00503-21_supp_1_seq4.pdf]

Table S1 The primers used in this study

Primer	Sequence (5'-3')
P1	TCGGACGGGAGAGTTTGTCTGTA
P2	GTCGTACAGATACAACCCAGACT
P3	GCAGCGAAGTTTATCAGATTG
P4	CCACACGGGAAGAGCGACGAATT
P5	ACTTTACTGGTGCTGCACAT
P6	CAAAATGATCCCCTCGTCAAC
P7	GCCCTGAGCGTCAAATTCCTT
P8	CGGCCCACAGAATGATGTCAC
oqxA-F	GGGGAAACATTCACCTGAC
oqxA-R	ACGGGAGACGAGGTTGGTA
oqxB-circle-F	CGCCATTCTGATCGTCGAGTTT
oqxA-circle-R	GAGCGCGGTCAGGTGAATGTTT

Table S2 Conjugation frequencies of cointegrate plasmids pC21-F1, pC21-F2, pC21-F3, and pC21-F4

Plasmid	From the parental strain to recipient *E. coli* C600			From the transconjugant to recipient *E. coli* J53		
	Mean	No.	Range	Mean	No.	Range
pC21-F1	7.1×10^{-9}	3	2.2×10^{-9}–1.1×10^{-8}	2.8×10^{-3}	3	1.2 × 10-3–5.4×10^{-3}
pC21-F2	2.6×10^{-7}	3	7.2×10^{-8}–6.1×10^{-7}	3.2×10^{-2}	3	1.9×10^{-2}–5.5×10^{-2}
pC21-F3	5.3×10^{-7}	3	2.4×10^{-7}–7.0×10^{-7}	2.7×10^{-2}	3	3.4×10^{-3}–6.8×10^{-2}
pC21-F4	1.0×10^{-7}	3	2.7×10^{-8}–2.0×10^{-7}	1.2×10^{-2}	3	1.5×10^{-3}–2.8×10^{-2}

Table S3 Fusion frequencies of non-conjugative plasmids pC21-2 and pHB37-2

Plasmid	IS*1294*	Target	Fusion frequency		
			Mean	No.	Range
pC21-2	IS*1294*	pC21-1	9.9×10^{-4}	3	5.9×10^{-4}–1.6×10^{-3}
pHB37-2	IS*1294*	pC21-1	2.1×10^{-4}	3	1.6×10^{-5}–3.4×10^{-4}

Table S4 Comparison of fusion frequencies of non-conjugative plasmids pC21-2 in *E. coli* C600 and the Δ*recA E. coli* C600

Plasmid	IS*1294*	Target	donor	Fusion frequency		
				Mean	No.	Range
pC21-2	IS*1294*	pC21-1	*E. coli* C600	3.0×10^{-4}	3	2.1×10^{-4}–4.4×10^{-4}
pC21-2	IS*1294*	pC21-1	Δ*recA E. coli* C600	4.8×10^{-6}	3	2.4×10^{-6}–9.1×10^{-6}

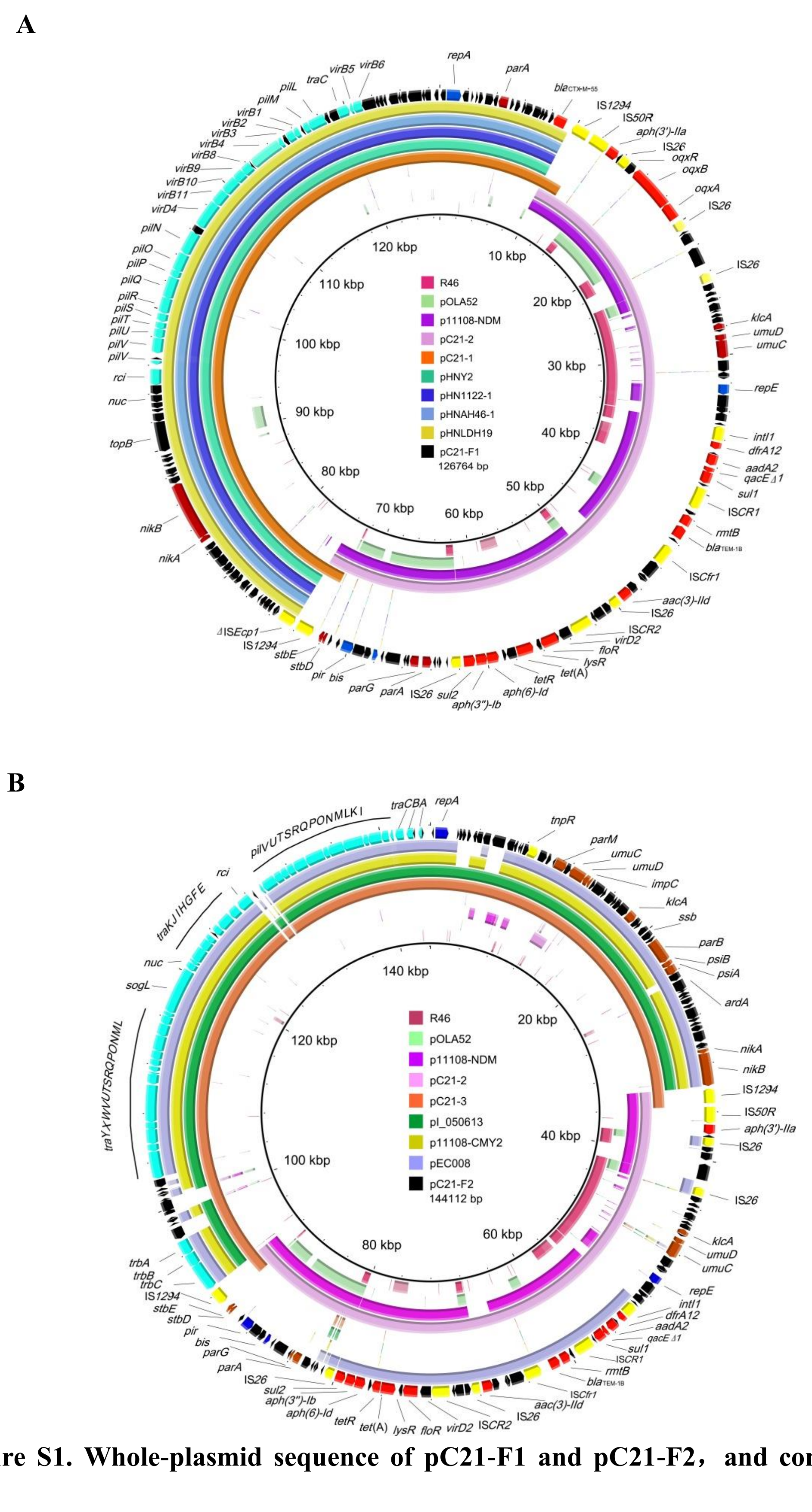

Figure S1. Whole-plasmid sequence of pC21-F1 and pC21-F2，and comparison of pC21-1，pC21-2, pC21-3 and the similar plasmids. Figure illustrates the insertion of pC21-2 into an IncI2 plasmids pC21-1 or an IncI1 plasmid pC21-3. Key features of

pC21-F1 and pC21-F2 are highlighted in different colors. Replicon genes are in blue; transfer associated genes are in cyan; resistance genes are in red; mobile elements are in yellow; stability associated genes are in maroon; and hypothetical proteins are in black. The fused regions are marked by dotted diagonal lines. (A) The outer ring comprises the CDSs of pC21-F1. Plasmids are pC21-F1, pC21-1, and pC21-2 in this study, and R46 (AY046276), pOLA52 (EU370913), pp1108-NDM (MG825381), pHNY2 (KF601686), pHN1122-1 (JN797501), pHNAH46-1 (KJ020576), pHNLDH19 (KM207012), and pEC008 (KY748190). (B) The outer ring comprises the CDSs of pC21-F2. Plasmids are pC21-F1, pC21-2, and pC21-3 in this study, and R46 (AY046276), pOLA52 (EU370913), pp1108-NDM (MG825381), pI_050613 (CP019215), p1108-CMY2 (MG825376), and pEC008 (KY748190).

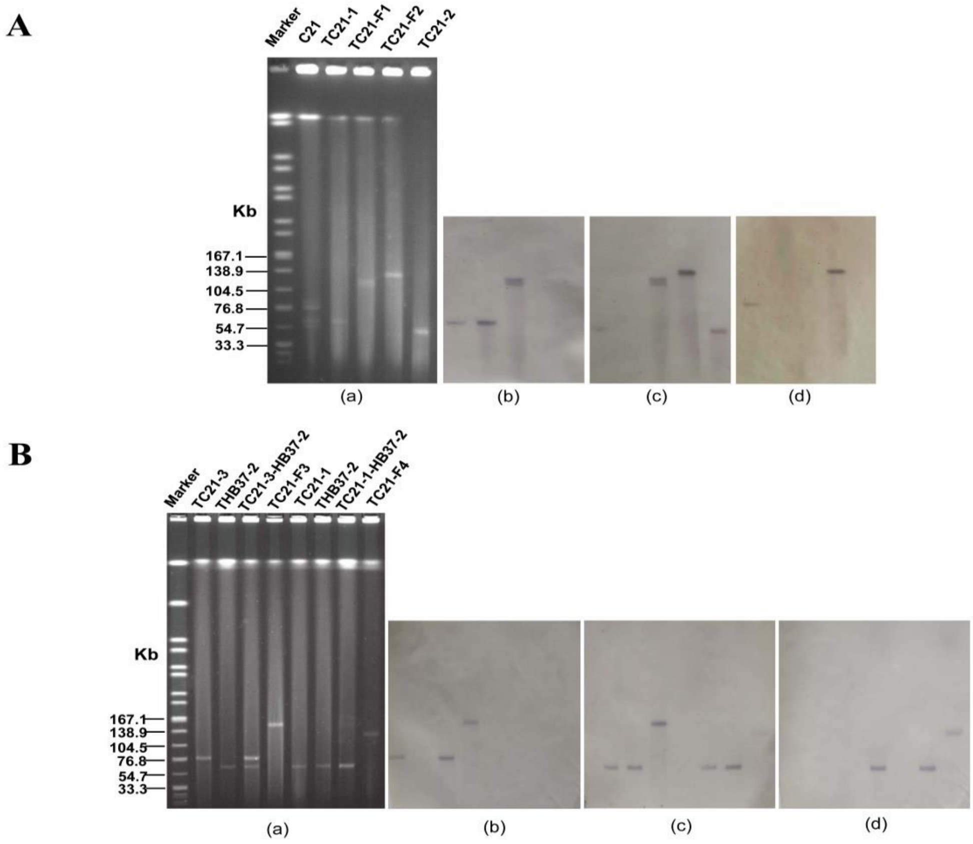

Figure S2. S1-PFGE and Southern hybridization. (A) S1-PFGE (a) and Southern hybridization (b, c, and d) of *E. coli* strain C21 and transconjugants TC21-1, TC21-F1, and TC21-F2, and the transformant TC21-2, with the *bla*$_{CTX-M-55}$, *rmtB*, and *trbA* as probes.

(B) S1-PFGE (a) and Southern hybridization (b, c and d) of the transconjugants TC21-F3 and TC21-F4, and transformants TC21-3, THB37-2, TC21-3-HB37-2, TC21-F3, TC21-1, and TC21-1-HB37-2, with the *trbA*, *rmtB*, and *bla*$_{\text{CTX-M-55}}$ as probes**.** Marker, *Salmonella Braenderup* H9812.

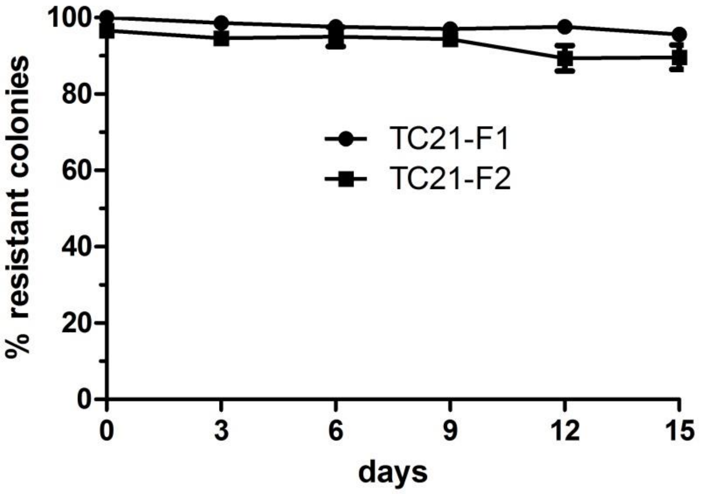

Figure S3. *In vitro* stability of fusion plasmids pC21-F1 and pC21-F2. Error bars represent the standard error of the mean of three independent experiments.

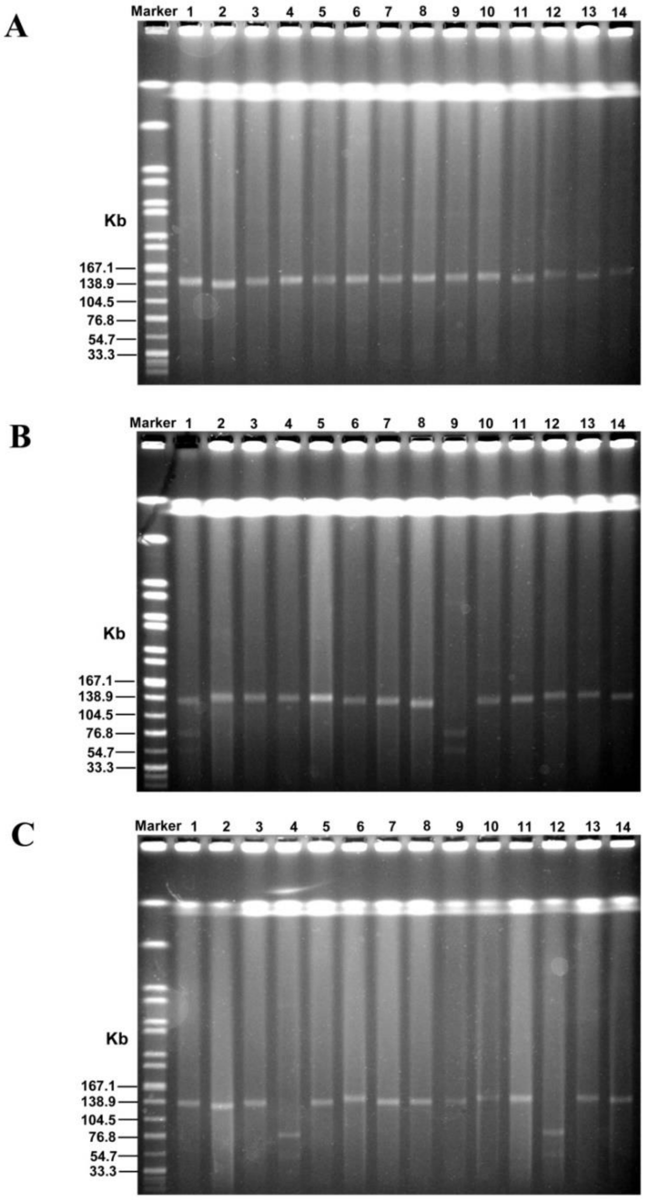

Figure S4. S1-PFGE analysis of plasmid profile in the transconjugant TC21-F2 colonies. (A) S1-PFGE of colonies at 9 days. (B) S1-PFGE of colonies at 12 days. (C) S1-PFGE of colonies at 15 days. No.1 to 14 were colonies randomly selected from TC21-F2 at 9, 12, 15 days, respectively. Marker, *Salmonella Braenderup* H9812.
